# RS12574989 and haplotype associated with α/β-chain imbalance and population HbA2 reduction

**DOI:** 10.1186/s12920-022-01333-6

**Published:** 2022-08-15

**Authors:** Qiyin Lin, Yingjun Xie, Xuan Zhong, Xiaofang Sun, Ding Wang

**Affiliations:** 1 Present Address: Department of Obstetrics and Gynecology, Guangdong Provincial Key Laboratory of Major Obstetric Diseases, The Third Affiliated Hospital of Guangzhou Medical University, No. 63 Duobao Road, Guangzhou, 510150 Guangdong China; 2 Guangdong Provincial Key Laboratory of Major Obstetric Diseases, The Third Affiliated Hospital of Guangzhou Medical University, Guangzhou, 510150 Guangdong China; 3 Medical Intensive Care Unit, Guangdong Women and Children Hospital, Guangzhou, 510000 Guangdong China

**Keywords:** α/β-chain imbalance, Thalassemia, Haemoglobin distribution, Single nucleotide polymorphisms, Haplotypes

## Abstract

Determining the associated relationship of genotype and phenomenon would benefit the understanding of disease and renew disease intervention means. 14,518 patients who underwent haemoglobin electrophoresis from June 2020 to December 2020 were enrolled in our study, and additional data including sex, age and routine blood examination results were collected. We focused on individuals with normal red blood cell indices and no common thalassemia pathogenic mutation and selected three groups for the following study: the control group (2.5% ≤ HbA_2_ ≤ 3.5%), the HbA_2_ under 2.5 group (HbA_2_ < 2.5%) and the HbA_2_ under 2.4 group (HbA_2_ < 2.4%). Four regions of β-globin regulation were sequenced. Statistical analysis was conducted to compare the collected information of the three groups and the genotype distributions in the control group and sequenced group. The HbA_2_ under 2.5 group was characterized by a majority of females and lower red blood cell counts and haemoglobin compared with the control group. There were genotypes associated with the grouping as the T of rs12574989 and TTTAGC of the haplotype were significantly increased in the HbA_2_ under 2.4 group and CTTAGC was significantly decreased in the HbA_2_ under 2.4 group. This study demonstrated that the genotypes of the population associated with HbA_2_ were reduced in southern China.

## Introduction

Thalassemia, as the natural selection against *Plasmodium falciparum* malaria [1], originates from certain warm areas, including the Mediterranean, Middle East, central Asia, India, and southern China; and has spread worldwide to be one of the most common genetic disorders [2, 3]. Guangdong located in the south of China with a high incidence of thalassemia [4], and over 10% of the population in this area are thalassaemia carriers [5]. According to the clinical spectrum, some thalassemia carriers have mild anaemia and are asymptomatic without needing regular transfusions, but they require occasional transfusions during some physiopathological state [6]. In addition, the offsprings of some carriers are at high risk of severe thalassemia [7]. Thalassemia screening to identify carriers will provide useful advice for people with special medical conditions and genetic counselling for healthy fertile individuals. In the clinical, the hematological examination results show that MCH (mean red blood cell hemoglobin) and MCV (mean red blood cell volume) are lower than the critical value, indicating that the risk of thalassemia is higher, and further gene diagnosis would be used for more accurate and efficient diagnosis for identification of the positive population. However, there were contradictory results as there is no clear genetic understanding of some odd haematological phenotypes during thalassemia screening. Genetic studies targeting specific populations with specific haematological phenotypes will enhance our understanding of the disease, even when determining a cure for the disease.

Thalassemia is a typical genetic disease with defects in haemoglobin [8]. The management of thalassemia depends on its clinical characteristics [9], which is a response to the genetic basis of Hb gene variation. There were two main human Hbs in normal adults, which were HbA and HbA2: HbA accounts for 96.5%-97.5% of Hbs and is the major protein. HbA consists of two α-and two β-globin chains and the rest of HbA2 consists of two α-and two δ-globin chains [10]. The balance of α- and β-globin chains comprises functional HbA, which is the molecular basis for Hb to perform its function [11].

Patients with thalassemia have genetic variants that cause abnormal α- or β-globin chains, which can be classified as α-thalassemia and β-thalassemia, as fail to produce these chains [12]. For those results of haemoglobin electrophoresis in α-thalassemia positive patients showed that HbA increased to more than 97.5%, but decreased to 96.5%, indicating a higher risk of β-thalassemia. The decreased in globin output of the β-globin caused by β-globin gene mutations was the genetic basis of β-thalassemia, which is one of the most important haemoglobinopathy in clinic [13].

The β-like globin genes are located in 11p15.5 [14], the expression of the human β-globin gene is switched after birth and replaces the foetal period expression gene γ-globin [15]. The β-globin gene is regulated by the 3’ enhancer [16] and 5’ locus control region (LCR), which is several kilobases upstream of named DNase I hypersensitive sites (HS) 2, HS3 and HS4 [17, 18]. According to the α/β-chain imbalance theory used to understand thalassaemia syndromes [19], it seems that the genetic polymorphism of regions regulating the β-globin gene might be associated with the high α-thalassemia risk of population.

To benefit disease understanding and therapy, it is necessary to discover the genetic characteristic of a specific population and understand the relationship between genotypes and phenotypes. In this study, we conducted a genetic study of patients with high screening risk according clinical haematology assay and a control (normal) population. First, we collected the basic information (age and sex), clinical haematological information (routine blood examination and haemoglobin electrophoresis) and thalassaemia genetic diagnosis results of patients who engaged in reproductive counselling. Then, an epidemiological study was used to select high α-thalassemia risk and thalassaemia genetic diagnosis negative populations for a genetic study of the regional polymorphisms of β-globin gene expression regulation including the β-globin 3’ enhancer, HS2, HS3 and HS4, as the β-globin gene is regulated by the 3’ enhancer [16] and 5’ locus control region (LCR), which is several kilobases upstream of named DNase I hypersensitive sites (HS) 2, HS3 and HS4 [17, 18]. Single nucleotide polymorphisms (SNPs) were identified in the 3’ enhancer, and we found that RS12574989 together with two haplotypes were associated with the clinical grouping. Collectively, we identified that genetic polymorphisms of the β-globin 3’ enhancer was associated with population characteristics of increased HbA and decreased HbA2. These results benefit the genetic understanding of β-globin regulation and β-thalassemia gene therapy.

## Materials and methods

### Study population

Our study was approved by the Academic Committee of the Third Affiliated Hospital of Guangzhou Medical University. From June 2020 to December 2020, 14,518 patients who underwent haemoglobin electrophoresis assays were enrolled in this study, and all of them signed informed consent before the study started. In order to eliminate the confounding factors, such as blood disease, age (especially new-borns) and blood transplantation, we selected patients from the reproductive department as the control and HbA_2_ under 2.5 group for further statistical analysis. The study items included sex, age, clinical examination and genotype.

### Haemoglobin electrophoresis

The peripheral blood of each subject was anticoagulated with EDTA for examination in this study. Whole blood was lysised using haemolysing solution. Haemoglobin electrophoresis was performed on a capillary electrophoresis instrument (Capillarys 2 Flex Piercing, Sebia, Evry, France) according to the manufacturer's instructions.

### Bloods routine examination

The haematological parameters of the peripheral blood were determined on an automatic haematology analyser (Sysmex XN-9000, Shanghai, China) according to the manufacturer's instructions.

### Genetic diagnosis of thalassemia

The genomic DNA of each individual was extracted from peripheral blood using a QIAamp DNA Blood Mini Kit (Cat# 51,104, Qiagen, Dusseldorf, Germany). The genetic diagnosis of thalassemia was performed using commercialized kits purchased from Yaneng Biosciences (Shenzhen, Guangdong, China). The deletions of α-globin were detected using gap polymerase chain reaction (Gap-PCR) for –SEA (NG_000006.1:g.26264_45564del19301), -α3.7 (NG_000006.1:g.34247_38050del) and -α4.2 (NG_000006.1:g.30681_(34619_34938)del). Point mutations were detected by DNA reverse dot blot hybridization. For α-globin, the mutations were ααCS (HBA2:c.427 T > C), ααQS (HBA2:c.377 T > C) and ααWS (HBA2:c.369C > G); and for β-globin, they were HBB:c.126_129delCTTT, HBB:c.316-197C > T, HBB:c.52A > T, HBB:c.-78A > G, HBB:c.79G > A, HBB:c.217dupA, HBB:c.130G > T, HBB:c.-79A > G, HBB:c.2 T > G, HBB:c.45dupG, HBB:c.85dupC, HBB:c.-82C > A, HBB:c.-80 T > C, HBB:c.92 + 1G > C, HBB:c.92 + 5G > C, HBB:c.94delC and HBB:c.-11_-8delAAACA.

### DNA segments sequencing

The DNA segments were amplified by PCR using AceTaq Master Mix (Cat# P412, Vazyme, Nanjing, China) with primers for HS2, HS3, HS4 and 3’ enhancer (Table 1). The sizes of the segments were confirmed by agarose electrophoresis, and Sanger sequence was using BigDye Terminator v3.1 Cycle Sequencing Kit (Cat #4,337,455, Thermo Fisher Scientific, CA, USA) according to the manufacturer's instructions. The mutations were determined by alignment with Genome Reference Consortium Human GRCh38.

**Table 1:** Primers for PCR β-globin regulation regions

Primer name	Base sequence (5'-3')	Size (bp)
HS4-F	CTGGACTTGTAATAGCTTTCTC	1099
HS4-R	CCTGGGTGAAGGTGAGAATTT	
HS3-F	AGAAGAGTCAAGCATTTGCCT	845
HS3-R	CTGGTTAGAAGGTTCTACTGG	
HS2-F	GTTGCAGTGAGCTGAGATC	618
HS2-R	CACATTCTGTCTCAGGCATC	
3’ enhancer-F	GAATGTGGGAGGTCAGTG	806
3’ enhancer-R	GTGGTTGATGGTAACACTATG	

### Statistical analysis

Quantitative results are expressed as the means ± SD and provide the range (minimum–maximum) while qualitative results are expressed as numbers of cases and ratios in tables. For quantitative data, ANOVA was used for multiple group comparisons, and the two-tailed Student's t-test was applied to compare the differences between the two groups. For qualitative data, the Pearson’s chi squared test was used for two-group distribution difference comparisons and odds ratios (ORs) with respective confidence interval (95% CI) calculations. Statistical analysis was performed using the SPSS 20.0 software (SPSS Inc., Chicago, IL, USA). P < 0.05 was regarded as a statistically significant difference. The linkage imbalance among the six alleles was analysed using the online SHESIS software (http://analysis.bio-x.cn/myAnalysis.php).

## Results

### Characteristics of high HbA, low HbA_2_ and normal MCV and MCH populations

**Fig. 1:**
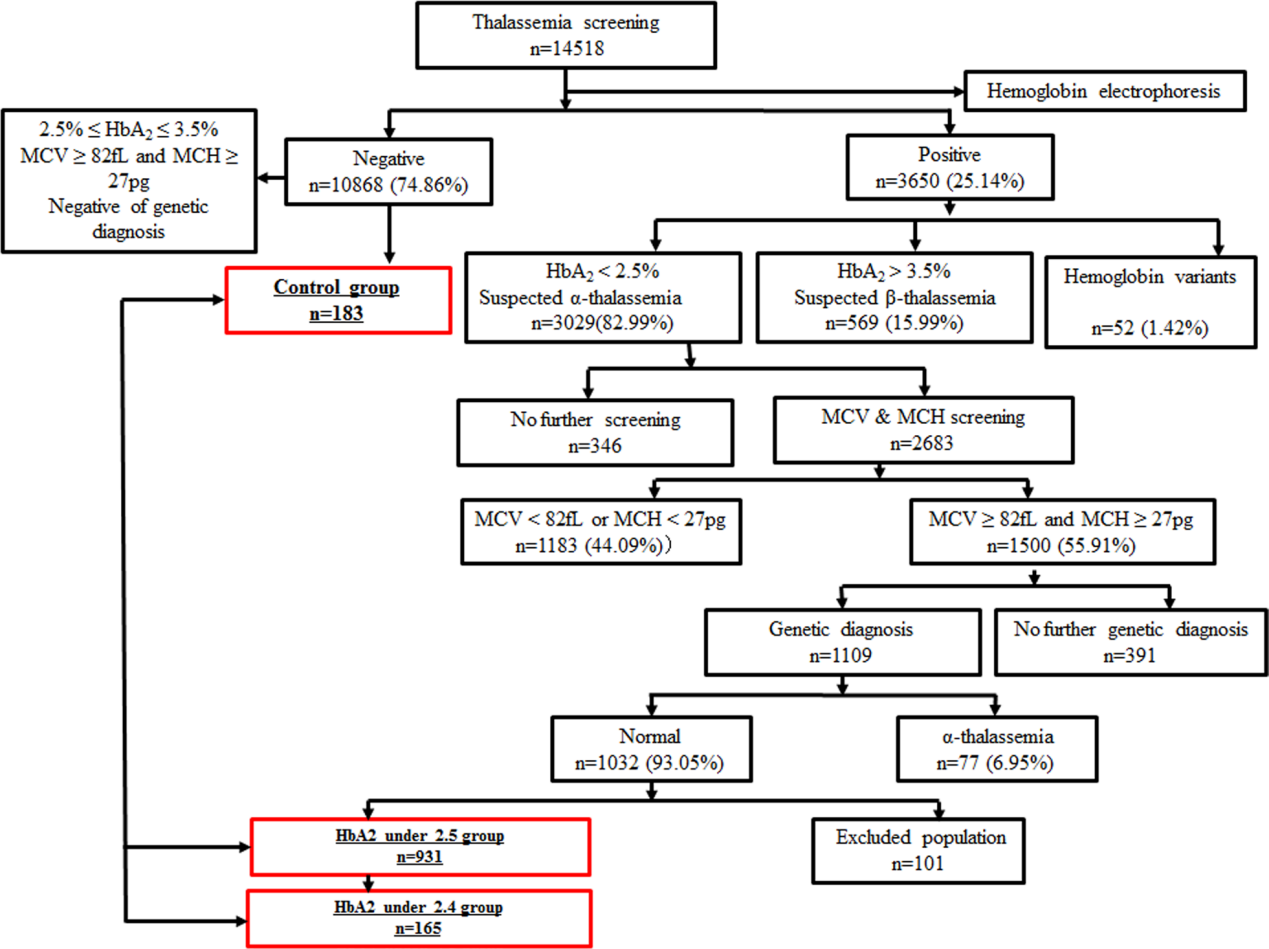
Flowchart of the selection of the study population

**Fig. 2:**
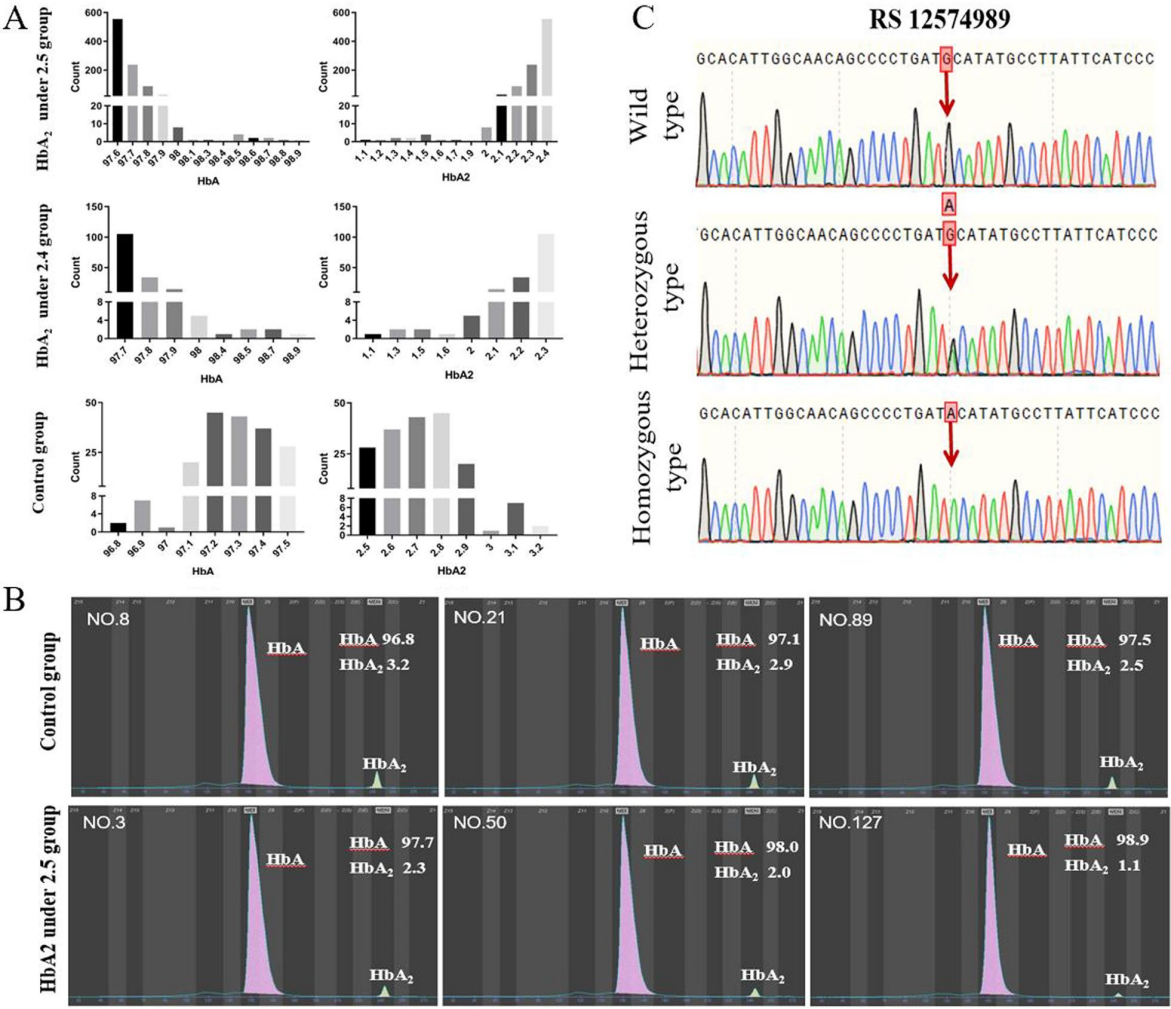
The SNP was identified associated with HbA_2_ reduction. **A** indicated distribution of HbA and HbA2 in different grouping. **B** indicated the haemoglobin electrophoresis of patients in the HbA2 reduce group and control (normal) group. **C** indicated genotype of rs12574989 C/T which was associated with HbA2 reduce grouping

In total, 14,518 individuals who underwent thalassemia screening by haemoglobin electrophoresis were included in this study (Fig. 1). The control group included 183 randomly selected individuals in the negative part of haemoglobin electrophoresis who possessed an HbA_2_ from 2.5% to 3.5% (Fig. 2. A and B), MCV ≥ 82 fL, MCH ≥ 27 pg and no thalassemia diagnose by genetic testing (Fig. 1). A total of 25.14% of individuals who underwent haemoglobin electrophoresis screening were positive for an abnormal HbA_2_ ratio or haemoglobin variants, and 82.99% of people were suspected to possess α-thalassemia with a low HbA_2_ (Fig. 1). In the HbA_2_ < 2.5% population, 55.91% of the people had normal MCV and MCH, 6.95% of the people were confirmed to possess α-thalassemia via genetic diagnosis and the rest were normal individuals (Fig. 1). The HbA_2_ under 2.5 group (Fig. 2. A and B) included 931 individuals excluding newborns, blood transplantation patients and patients with haematological malignancies, who shared similar MCV, MCH and thalassemia genetic diagnosis results with the control group but obtained an HbA_2_ below 2.5% (Table 2).

**Table 2:** Characteristics of the HbA2 reduce group and control group

		HbA2 under 2.5 group (n = 931)	HbA2 under 2.4 group (n = 165)	Control group (n = 183)	*P* value		
					HbA2 under 2.5 (n = 934) VS. HbA2 under 2.4 (n = 165)	HbA2 under 2.5 (n = 934) VS. Control (n = 183)	HbA2 under 2.4 (n = 165) VS. Control (n = 183)
Age	Mean ± SD	32.87 ± 5.81	33.21 ± 5.67	32.46 ± 5.08			
	Range	21–54	22–49	22–46	0.487	0.377	0.1953
Gender [n(%)]	Male	347(37.15)	50(30.3)	85(46.45)			
	Female	587(62.85)	115(69.7)	98(53.55)	0.065	0.025	< 0.001
MCV [fL]	Mean ± SD	88.10 ± 3.30	88.48 ± 3.39	88.34 ± 3.55			
	Range	82–99.8	82.2–97.8	82–99.4	0.17	0.362	0.7118
MCH [pg]	Mean ± SD	30.12 ± 1.28	30.08 ± 1.24	30.24 ± 1.14			
	Range	27–34.4	27.1–33.7	27.7–33.9	0.735	0.213	0.2007
MCHC [g/dL]	Mean ± SD	34.19 ± 0.99	34.04 ± 0.84	34.27 ± 0.83			
	Range	31.2–38.1	31.7–36.3	32.1–36.5	0.068	0.325	0.0112
RBC [10^12/L]	Mean ± SD	4.64 ± 0.5	4.62 ± 0.46	4.74 ± 0.47			
	Range	2.94–6.17	3.24–6.03	3.8–5.84	0.517	0.011	0.011
Hb [g/dL]	Mean ± SD	13.97 ± 1.51	13.9 ± 1.38	14.34 ± 1.4			
	Range	8.7–18	9.9–18	11.5–17.5	0.573	0.003	0.0037
RDW [%]	Mean ± SD	12.6 ± 0.74	12.58 ± 0.83	12.44 ± 0.6			
	Range	10.9–18.4	10.9–18.4	11.2–15.5	0.831	0.007	0.0646

### Significant differences in sex and routine blood indices were found between the HbA2 under 2.5 group and the control group.

165 individuals in the HbA_2_ under 2.5 group and HbA higher than 97.6% were randomly selected and included in the HbA_2_ under 2.4 group (Fig. 2. A). The distribution of HbA and HbA2 in the HbA_2_ under 2.4 group was similar to that in the HbA_2_ under 2.5 group (Fig. 2. A and B). The basic information and clinical haematological information between the HbA_2_ under 2.5 group (n = 931), HbA_2_ under 2.4 group (n = 165) and control group (n = 183) were statistically analysed. There were no significant differences between the HbA_2_ under 2.5 group and HbA_2_ under 2.4 group (Table 2). The HbA_2_ under 2.5 group and HbA_2_ under 2.4 group had a high proportion of females compared with the control group, and the sex ratio of the control group was near 1:1 (Table 2). There was no significant difference of MCV and MCH in groups (Table 2). There were consistent trends as a reduce of MCHC and RDW in HbA_2_ reduce groups compare with control group without statistical difference (Table 2). There were consistent results in RBC and Hb as significant decreases in RBC (P value < 0.05) and Hb (P value < 0.01) were observed in the HbA_2_ reduce group compared to the control group (Table 2).

### Genotype associated with clinical grouping

To determine the gene variations of β-globin regulation, four segments were sequenced for genotyping in a total of 20 individuals (10 for the HbA_2_ reduce group and 10 for the control group). Because there was no variation found in HS2, HS3 and HS4, further study was focused on the 3’ enhancer. There were no deletions or insertions, but six single nucleotide polymorphisms were found: rs12574989, rs7110263, rs10837631, rs78928216, rs10837630 and rs11036351. In the comparison of the HbA_2_ reduce group and control group, a significant increase in the rs12574989 mutation type (heterozygous type-CT and homozygous type-TT, Fig. 2. C) was found in the HbA_2_ under 2.4 group, but no difference was found in the other loci (Table 3). Statistical analysis of six SNPs formed a haplotype prompt that CTTAGC significantly decreased and TTTAGC significantly decreased in the HbA_2_ reduce group compared with the control group (Table 3).

**Table 3:** Statistical analysis of the distribution of SNPs and haplotypes in the HbA2 reduce group and control group

	HbA2 under 2.4 group (n = 165)	Control group (n = 183)	OR[95%CI]	*P* value
*rs 12574989 C/T*				
CC	102(0.618)	159(0.869)	1	
CT	57(0.345)	23(0.126)	3.863[2.242–6.658]	
TT	6(0.036)	1(0.005)	9.353[1.110–78.826]	< 0.001
CT + TT	63(0.382)	24(0.131)	9.353[1.847–2.268]	< 0.001
C	261(0.791)	341(0.932)		
T	69(0.209)	25(0.068)	17.613[1.007–1.259]	< 0.001
*rs 7110263 T/G*				
TT	44(0.267)	50(0.273)	1	
GT	84(0.509)	98(0.536)	0.469[0.313–0.702]	
GG	37(0.224)	35(0.191)	0.365[0.212–0.630]	0.746
G	158(0.479)	168(0.459)		
T	172(0.521)	198(0.541)	1.083[0.804–1.459]	0.602
*rs 10837631 T/A*				
TT	82(0.497)	82(0.448)	1	
AT	66(0.400)	84(0.459)	0.786[0.504–1.226]	
AA	17(0.103)	17(0.093)	1[0.478–2.093]	0.54
A	100(0.303)	118(0.322)		
T	230(0.697)	248(0.678)	0.914[0.663–1.260]	0.582
*rs 78928216 A/C*				
AA	109(0.661)	135(0.738)	1	
AC	51(0.309)	46(0.251)	0.188[1.373–0.857]	
CC	5(0.030)	2(0.011)	0.182[3.096–0.589]	0.183
A	269(0.815)	316(0.863)		
C	61(0.185)	50(0.137)	0.698[0.464–1.049]	0.083
*rs 10837630 G/C*				
GG	45(0.273)	51(0.279)	1	
CG	82(0.497)	96(0.525)	0.898[0.968–0.589]	
CC	38(0.230)	36(0.197)	0.563[1.196–0.652]	0.741
C	158(0.479)	168(0.459)		
G	172(0.521)	198(0.541)	1.083[0.803–1.459]	0.602
*rs 11036351 C/T*				
CC	110(0.667)	136(0.743)	1	
CT	51(0.309)	46(0.251)	0.19[1.371–0.856]	
TT	4(0.024)	1(0.005)	0.155[4.945–0.545]	0.143
C	271(0.821)	318(0.869)		
T	59(0.179)	48(0.131)	0.693[0.458–1.049]	0.082
*Haplotype*				
C G A A C C	96(0.291)	112(0.306)	0.906 [0.653–1.257]	0.555
C G T C C T	57(0.173)	46(0.126)	1.425 [0.935–2.173]	0.098
C T T A G C	101(0.306)	170(0.464)	0.487 [0.356–0.668]	< 0.001
T T T A G C	69(0.209)	24(0.066)	3.710 [2.267–6.072]	< 0.001

## Discussion

According to clinical diagnosis and management differences, thalassemia can be divided into three levels: transfusion-dependent thalassemia (TDT), nontransfusion-dependent thalassemia (NTDT) and minor thalassemia [12]. Thalassemia has affected individuals since birth with clinical criteria including anaemia, reduced Hb, significant hepatosplenomegaly, retarded growth and height and frequent intercurrent infections [12, 20]. The effective management of thalassemia could improve clinical symptoms but not provide fundamental solutions, such as blood transfusions, a splenectomy, iron chelation, and even stem cell transplantation [12, 21]. In clinical, the commercial kit could provide over ninty-persents conformed genetic thalassemia diagnosis report, but there was not certain genetic explanation for some clinical features. To completely avoid severe thalassemia, it is necessary to screen thalassemia carriers that could affect offspring [7] and study the pathogenesis-related genetics to produce available treatments.

Thalassemia screening aimed to identify individuals who were potential pathogenic mutation carriers using red blood cell indices and haemoglobin analysis. The widely used indices for screening red blood cell indices were Hb, MCV and MCH as values lower than the cut-offs (11 g/dL for Hb, 27 pg for MCH and 82 fL for MCV) prompt high risk [21, 22]. However, for clinical usage, the cut-offs were slightly adjusted for local people as we defined high risk as Hb < 11.5 g/dL and MCV < 82 fL. Hb analysis could be conducted by capillary electrophoresis, which is an approved platform of Hb analysers [23], and used to identify if there are some Hb variants and determine the ratio of HbA and HbA_2_. In our study, the majority of the HbA_2_ < 2.5% population (82.99%) was Hb positive, and more than half (55.91%) of these patients had a normal MCV and MCH. In this population, a small percentage (6.95%) of patients were diagnosed as thalassemia if they were diagnosed as α-thalassemia without any β-thalassemia, the majority (93.05%) of these patients were not diagnosed with any pathogenic mutations. It is worth noting that the individuals in our HbA_2_ reduce group are those with normal heredity and erythrocyte index, and their HbA_2_ proportion has decreased. Statistical analysis showed that the majority of this population was female and had a low RBC and Hb compared with the control group.

The theory of α/β-chain imbalance refers to that the pathogenic mutation leads to the restriction of one globin expression and induces the excess of another globin, which directly leads to ineffective erythropoiesis, and further leads to iron overload, anemia and organ damage [19]. Like β-thalassemia, α-thalassemia is caused by a disease-causing genotype that makes defective production of α globin. The typical haematology characteristics of α-thalassemia patients were up-regulation of HbA and down-regulation of HbA_2_. Multi genetic reasons leading this clinical phenomenon, beside the α-globin abnormal, multi genetic reasons leading this clinical phenomenon. We focus on genetic variation analysis of β-globin regulation regions.

Genetic variants in functional regions are associated with gene expression and disease pathological progress [24]. The β-globin regulation regions, including HS regions [25, 26] and their 3’ enhancers [16], were found decades ago. As their function was proven in cell cultures and in transgenic mice [17], an engineered lentiviral product was created for β-thalassemia treatment, which could spatially and temporally mimic functional β-globin expression [27]. Furthermore, the clinical symptoms of β-thalassemia were effectively alleviated by transplantation of autologous haematopoietic stem cells (HSCs), in which lentiviruses transferred normal and functional β-globin [28]. The functional regions of β-globin regulation in the lentiviral product were detected in our study. HS was a conserved region without any variants, and six SNPs were found in the 3’ enhancer. Only one SNP (rs12574989) was associated with the grouping. As C turned to T, the CpG island of the wild type was destroyed by this mutation. CpG islands are classical methylation sites. This is consistent with our hypothesis that the loss of wild type results in increased gene expression. Furthermore, the two haplotypes, which were generated because of SNP variation, were also associated with the grouping. CTTAGC was significantly decreased and TTTAGC was increased in HbA_2_ under 2.4 group.

The variation of RS12574989 was different in regions base on the database of dbSNP (https://www.ncbi.nlm.nih.gov/snp/), and the ALFA project and 1000Genomes reported similar results, the alt allele ‘T’ was under 5% in African (0.15%), Europe (2.68%) and American (1.3%). The top occurrence was East Asian (10.91%) and South Asian (8.7%), according the study of 1000Genomes (https://www.ncbi.nlm.nih.gov/snp/rs12574989#frequency_tab). However, there were seldom published literature covered the individual pathophysiological phenomenon associated with variant RS12574989 and haplotypes. In the current study, it is identified that significantly increased SNP ‘T’ of rs12574989 was only 20.9% of individuals with the HbA_2_ under 2.4 group, that means the relationship of the clinical phenomenon and genetic was asscoiated but not causal, and there were other potential reasons to lead the HbA abnormal. However, the HbA level of the individuals who got ‘T’ variation were 97.5% to 98%, this prompt the genetic variation might related to the clinical phenomenon. Although the HbA_2_ under 2.4 group is 165, they were sufficient to conclude with clinical and genetic features in southern China. Firstly, the HbA_2_ under 2.4 group was the representative of the HbA_2_ under 2.5 (n = 931), at which share consistent clinical information and distribution of HbA and HbA2. Secondly, the HbA_2_ reduce group was 6.4% (931/14518) of the total, as there were 14,518 thalassemia screening people were included in this study. Further large scale genetic testing to find out more CTTAGC haplotype and comparison study for the interplay between the genetic and haematological phenotype was needed.

In the present study, we demonstrated specific genotype enrichment in a HbA_2_ reduced population, but the influence on disease pathological progress was unknown. Further studies are needed to define if the single base difference leading the gene expression difference and the involved mechanism, such as epigenetic.

## Conclusion

In summary, our study focused on a population with normal red blood cell indices and no common pathogenic mutation of the α- and β-globin genes but a reduced HbA2 ratio. We demonstrated that the population was primarily female and had low RBC and Hb compared with the control group, and a genetic variation in the functional region of the β-globin gene was associated with this population. Further functional studies are needed to define the biological function of haplotypes if they cause β-globin expression changes, which would benefit gene therapy strategy designs.

## Data Availability

The datasets presented in this study can be found in online repositories. The names of the repository/repositories and accession number(s) can be found below: ClinVar SCV001809802.
